# Associations Between Sexual Orientation and Overall and Site-Specific Diagnosis of Cancer: Evidence From Two National Patient Surveys in England

**DOI:** 10.1200/JCO.2017.72.5465

**Published:** 2017-09-25

**Authors:** Catherine L. Saunders, Catherine Meads, Gary A. Abel, Georgios Lyratzopoulos

**Affiliations:** Catherine L. Saunders and Georgios Lyratzopoulos, Cambridge Centre for Health Services Research, University of Cambridge; Catherine Meads, Anglia Ruskin University, Cambridge; Gary A. Abel, University of Exeter Medical School, Exeter; and Georgios Lyratzopoulos University College London, London, United Kingdom.

## Abstract

**Purpose:**

To address gaps in evidence on the risk of cancer in people from sexual minorities.

**Patients and Methods:**

We used data from 796,594 population-based English General Practice Patient Survey responders to explore the prevalence of self-reported diagnoses of cancer in the last 5 years among sexual minorities compared with heterosexual women and men. We analyzed data from 249,010 hospital-based English Cancer Patient Experience Survey responders with sexual orientation as a binary outcome, and International Classification of Diseases, Tenth, Revision, diagnosis as covariate—38 different common and rarer cancers, with breast and prostate cancer as baseline categories for women and men, respectively—to examine whether people from sexual minorities are over- or under-represented among different cancer sites. For both analyses, we used logistic regression, stratified by sex and adjusted for age.

**Results:**

A diagnosis of cancer in the past 5 years was more commonly reported by male General Practice Patient Survey responders who endorsed gay or bisexual orientation compared with heterosexual men (odds ratio [OR], 1.31; 95% CI, 1.15 to 1.49; *P* < .001) without evidence of a difference between lesbian or bisexual compared with heterosexual women (OR, 1.14; 95% CI, 0.94 to 1.37; *P* = .19). For most common and rarer cancer sites (30 of 33 in women, 28 of 32 in men), the odds of specific cancer site diagnosis among Cancer Patient Experience Survey respondents seemed to be independent of sexual orientation; however, there were notable differences in infection-related (HIV and human papillomavirus [HPV]) cancers. Gay or bisexual men were over-represented among men with Kaposi’s sarcoma (OR, 48.2; 95% CI, 22.0 to 105.6), anal (OR, 15.5; 95% CI, 11.0 to 21.9), and penile cancer (OR, 1.8; 95% CI, 0.9 to 3.7). Lesbian or bisexual women were over-represented among women with oropharyngeal cancer (OR, 3.2; 95% CI, 1.7 to 6.0).

**Conclusion:**

Large-scale evidence indicates that the distribution of cancer sites does not vary substantially by sexual orientation, with the exception of some HPV- and HIV-associated cancers. These findings highlight the importance of HPV vaccination in heterosexual and sexual minority populations.

## INTRODUCTION

Over the last 30 years, there has been substantial research efforts in the area of the health of sexual minority populations; however, this research has focused on sexually transmitted diseases, particularly HIV,^1,2^ and little is known about how cancer risk varies among sexual minorities compared with heterosexual populations.^2-4^ This contrasts with the increasing disease burden that is associated with cancer, which is currently the leading cause of death in high-income countries,^5^ and, after mental health services and circulatory diseases, cancer services make up the third largest category of spending in contemporary health care systems.^6^

Approximately four of 10 cancers are attributable to lifestyle and environmental risk factors, including smoking, alcohol consumption, dietary factors, occupational exposures, and sexual and reproductive history.^7-9^ Disparities in cancer incidence among people from sexual minorities compared with heterosexual populations are likely. Lifetime exposure to risk factors among sexual minority and heterosexual populations is likely to differ—for example, smoking initiation is higher among lesbian, gay, and bisexual young people in both the United Kingdom^10^ and the United States.^11^ Hormonal factors are also likely to be important for some cancers; child bearing and the use of oral contraceptives are associated with a risk of female breast and ovarian cancer,^12^ and lesbian and heterosexual women are differently exposed to these two factors.^13^ In addition, HIV prevalence in the United Kingdom is 0.2%, but among men who have sex with men (age 15 to 44 years), it is 5%^14^; immune deficiency is associated with an increased risk of several cancers.^15,16^ Approximately 4.8% of cancer diagnoses worldwide in 2008 were attributable to human papillomavirus (HPV), with differences in exposure burden in men and women.^17^ Understanding how the risk of cancer varies by sexual orientation is therefore of particular importance to help understand where best to target preventive efforts.

In the United States, the Institute of Medicine in 2011 called for research investment in the health of sexual minorities, including basic epidemiologic research, which highlighted the lack of relevant evidence.^18^ Similarly, ASCO has called for research on sexual orientation–related disparities and increased data collection.^19^ In the United Kingdom. cancer charities have highlighted the continuing limitations of data on lesbian, gay, and bisexual people with cancer.^20^

It is usually difficult to study sexual orientation in population health and epidemiologic studies, primarily because this information is simply not known or not collected.^19^ Nonetheless, provided that items on sexual orientation are included, patient experience surveys provide unique opportunities for acquiring insight into the risk of cancer in sexual minorities.^21^

Against this background, we used data from two English patient surveys, the General Practice Patient Survey (GPPS) and the Cancer Patient Experience Survey (CPES), to examine two research questions: Do women and men from sexual minorities report a cancer diagnosis in the previous 5 years more or less frequently than heterosexual women and men? And, among recently treated survivors of cancer, is there variation between cancer sites in the proportion of men and women who report gay, lesbian, or bisexual sexual orientation?

## PATIENTS AND METHODS

### Data

GPPS is a national survey of the patient experience of primary care and is sent by postal mail to approximately 2.7 million patients in England age ≥ 18 years who have been continuously registered with a general practice for at least 6 months, with a respondent sample of approximately 1 million (response rate approximately 37%). A stratified sample of patients was drawn from the practice list of each general practice in England, with oversampling of patients from practices that were known from prior surveys to have low response rates. Full details are published in the technical report.^22^ Data from 2011/2012 were used in this analysis. Data from GPPS have been previously used to describe the patient experience, health-related quality of life, and health service utilization of sexual minorities.^23,24^

CPES is a separate survey of recently treated survivors of cancer^25^ that is sent annually to all patients age ≥ 16 years who were treated for cancer in a National Health Service hospital in England during a 3-month period. Anonymous data from 2010, 2011/2012, 2013, and 2014 were obtained via the UK Data Archive, and full survey details are available.^26-29^

This study involved the secondary analysis of previously collected anonymous data, for which formal ethical approval is not required.^30^ All cancer diagnoses are included in this analysis; however, cells counts of fewer than six individuals are suppressed in reporting, which is in line with best practice.^31^

### Overall and Site-Specific Diagnosis of Cancer

In the GPPS, respondents are asked “Which, if any, of the following medical conditions do you have?,” with 16 response options, including “Cancer in the last 5 years,” plus “None of these conditions” and “I would prefer not to say”. “Prefer not to say” responses and responses for which no options were ticked were coded as missing for this analysis. No additional detail about the nature of the cancer diagnosis—that is, in relation to cancer site—is available in this survey.

In CPES, patients were identified for inclusion in the survey sampling frame when the main hospital record for inpatient or outpatient treatment recorded an International Classification of Diseases, Tenth Revision cancer diagnosis code for inpatient or outpatient cancer treatment. In line with previous research,^32^ but with the addition of Kaposi’s sarcoma, we included 38 common and rarer cancer site groups in the analysis (Table A8, online-only).

### Sexual Orientation

Survey questions were used to identify respondents’ sexual orientation in both surveys. In GPPS, “Which of the following best describes how you think of yourself?” had the following possible responses: “Heterosexual/straight,” “Gay/Lesbian,” “Bisexual,” “Other,” or “I would prefer not to say”. In CPES, “Which of the following best describes your sexual orientation?” has the following possible responses: “Heterosexual/straight (opposite sex),” “Bisexual (both sexes),” “Gay or Lesbian (same sex),” “Other,” of “Prefer not to answer.”

### Demographic Information

In GPPS, survey responses for age 18 to 24 years, then 10-year age groups to age ≥ 85 years, gender, and ethnicity in five groups (ONS2011) were used. In CPES, hospital record recorded age and gender (as these are more complete), and survey-reported ethnicity in six groups (ONS2001) were used. For both surveys, the Index of Multiple Deprivation—a small geographic area measure of socioeconomic deprivation, derived from respondents’ postcodes—was used and divided into five groups by using quintile-defining cut points.^33^

### Analysis

In all analyses, women and men are considered separately, and all respondents—both those from sexual minorities and those who report heterosexual sexual orientation—are included. GPPS survey data are provided with weights that account for design and nonresponse^22^; therefore, descriptive analysis is presented for weighted data. For CPES, as all cancer cases within the sampling period were selected, design weights are not applicable, and only unweighted data are presented.

For our first analysis, using data from GPPS we performed logistic regression to examine variations in report of cancer diagnosis in the last 5 years by sexual orientation, including a test of any difference between gay or lesbian and bisexual individuals.

For the second analysis, we used data from recently treated survivors of cancer who responded to CPES with sexual orientation as a binary outcome—grouping gay or lesbian and bisexual respondents—and cancer site as covariate. This analysis aimed to reveal overall patterns of variation between cancer sites in the proportion of men and women who report gay, lesbian, or bisexual sexual orientation. For a particular cancer site, higher or lower odds reflect differences in cancer diagnosis among people from sexual minorities compared with the reference site (breast in women, prostate in men). Our choice of these reference sites was based on analytical considerations—that is, because they were the cancers with the largest sample size, which allowed the most precise comparisons. In addition, from these models, we predicted the adjusted percentage of women and men with cancer of a particular site who were expected to report lesbian or bisexual or gay or bisexual sexual orientation should they have the same age composition as all included survey responders with this diagnosis (this percentage is also known as a recycled prediction).

Adjusting for ethnicity, deprivation, survey wave (CPES), and GP practice (GPPS) or hospital of treatment (CPES; using a random effect for organization) had a minimal effect on coefficients for cancer site, and, given this, to reduce the amount of missing observations as a result of incomplete information on deprivation and ethnicity, these variables were dropped from the main analyses, which are only adjusted for age (Appendix Tables A1 to A3, online only).

### Supplementary Analyses

We explored a series of sensitivity analyses. First, we considered each response option to the sexual orientation question separately—that is, “Gay/Lesbian,” “Bisexual,” “Other,” “I would prefer not to say,” and missing responses were compared with the response, “Heterosexual/straight,” to examine potential associations between our outcomes and patient groups other than those that endorsed heterosexual, gay/lesbian, and bisexual response options. Second, for CPES analysis, we also restricted the analysis to people who had been diagnosed with cancer in the past year to consider a population that more closely represented incident cancer cases^34^ and to explore the potential impact of the same respondents being included across survey waves.

## RESULTS

Among 796,594 respondents from the population-based GPPS sampling frame, 32,437 of all respondents (3.0%) reported cancer in the past 5 years. Of the 12,177 respondents (2.1%) from sexual minorities, 361 (1.9%) reported cancer in the past 5 years. Among the 240,010 recently treated survivors of cancer who responded to CPES between 2010 and 2014, there were 2,199 respondents (0.9%) who endorsed a sexual minority orientation.

Before adjustment for age, people from sexual minorities are less likely to report cancer in the past 5 years, as, on average, people who report nonheterosexual sexual orientation are younger than those who report heterosexual sexual orientation.^23^ After adjusting for age, GPPS data provided no evidence of a difference between heterosexual and lesbian or bisexual women (odds ratio [OR], 1.14; 95% CI, 0.94 to 1.37; *P* = .19); however, gay or bisexual men were more likely to report cancer in the past 5 years than heterosexual men (OR, 1.31; 95% CI, 1.15 to 1.50; *P* < .001), with evidence of a difference between gay (OR, 1.45; 95% CI, 1.24 to 1.69) and bisexual men (OR, 1.00; 95% CI, 0.78 to 1.30; Table 2).

Although lesbian or bisexual women represented 0.7% of all female CPES responders (any cancer site), they represented 2.3% (adjusted, 2.1%) of women with oropharyngeal cancer, 2.0%(adjusted, 1.1%) with Hodgkin lymphoma, and 1.3% (adjusted, 0.7%) of women with cervical cancer. In the same way, although gay or bisexual men represented 1.1% of all male CPES responders (any cancer site), they made up 46.4%(adjusted, 35.4%) of men with Kaposi’s sarcoma, 17.3% (adjusted, 15.7%) of men with anal cancer, 2.9% (adjusted, 1.4%) of men with Hodgkin lymphoma, 2.5% (adjusted, 0.9%) of men with testicular, and 2.5% (adjusted, 1.6%) thyroid cancers. Again, because endorsing a sexual minority orientation in the two surveys was more common in younger patients,^23^ crude figures are confounded by age, with the crude proportions of men and women from sexual minorities higher for cancer sites where diagnosis among young people is more common—for example, cervical and testicular cancer, and Hodgkin Lymphoma (Tables 1 and 3).

**Table 1.**
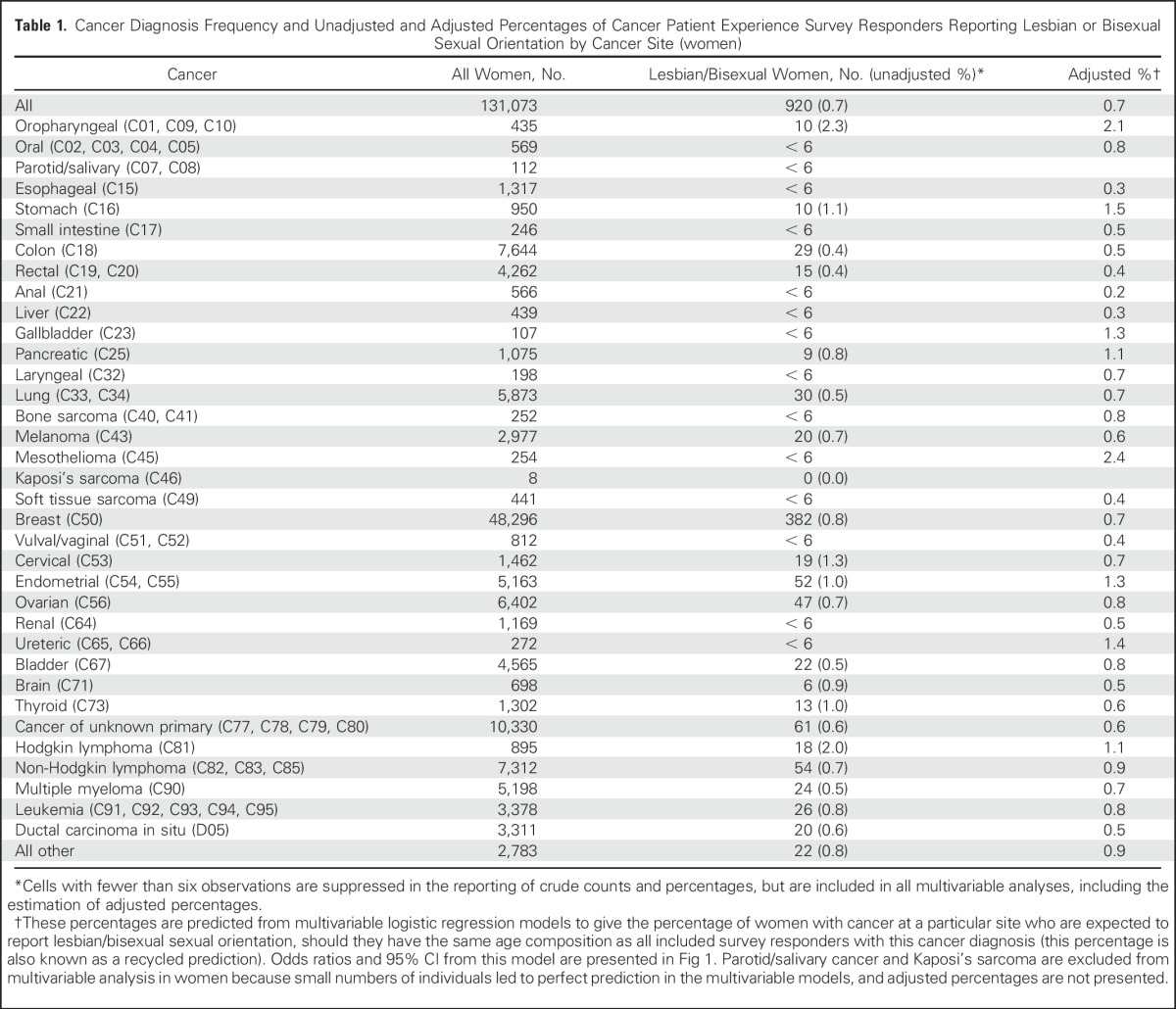
Cancer Diagnosis Frequency and Unadjusted and Adjusted Percentages of Cancer Patient Experience Survey Responders Reporting Lesbian or Bisexual Sexual Orientation by Cancer Site (women)

**Table 2.**
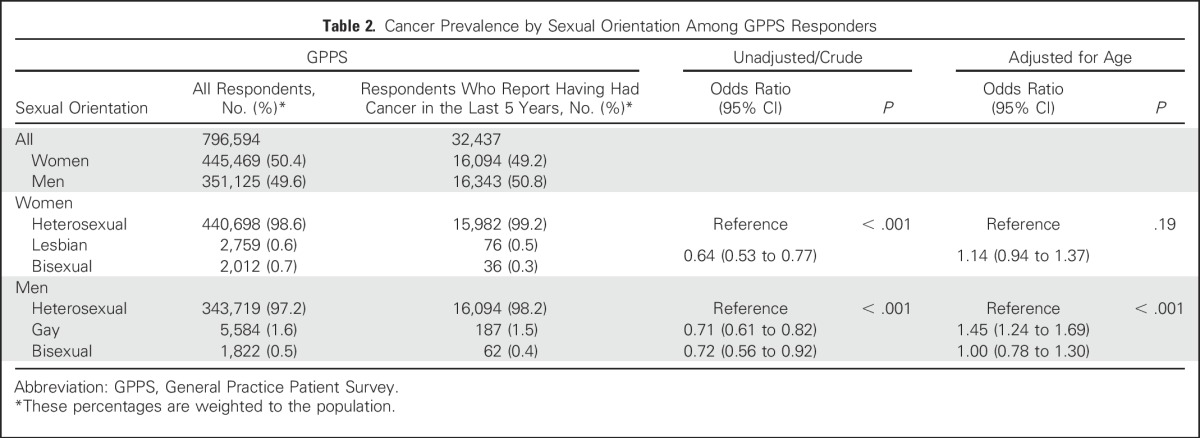
Cancer Prevalence by Sexual Orientation Among GPPS Responders

**Table 3.**
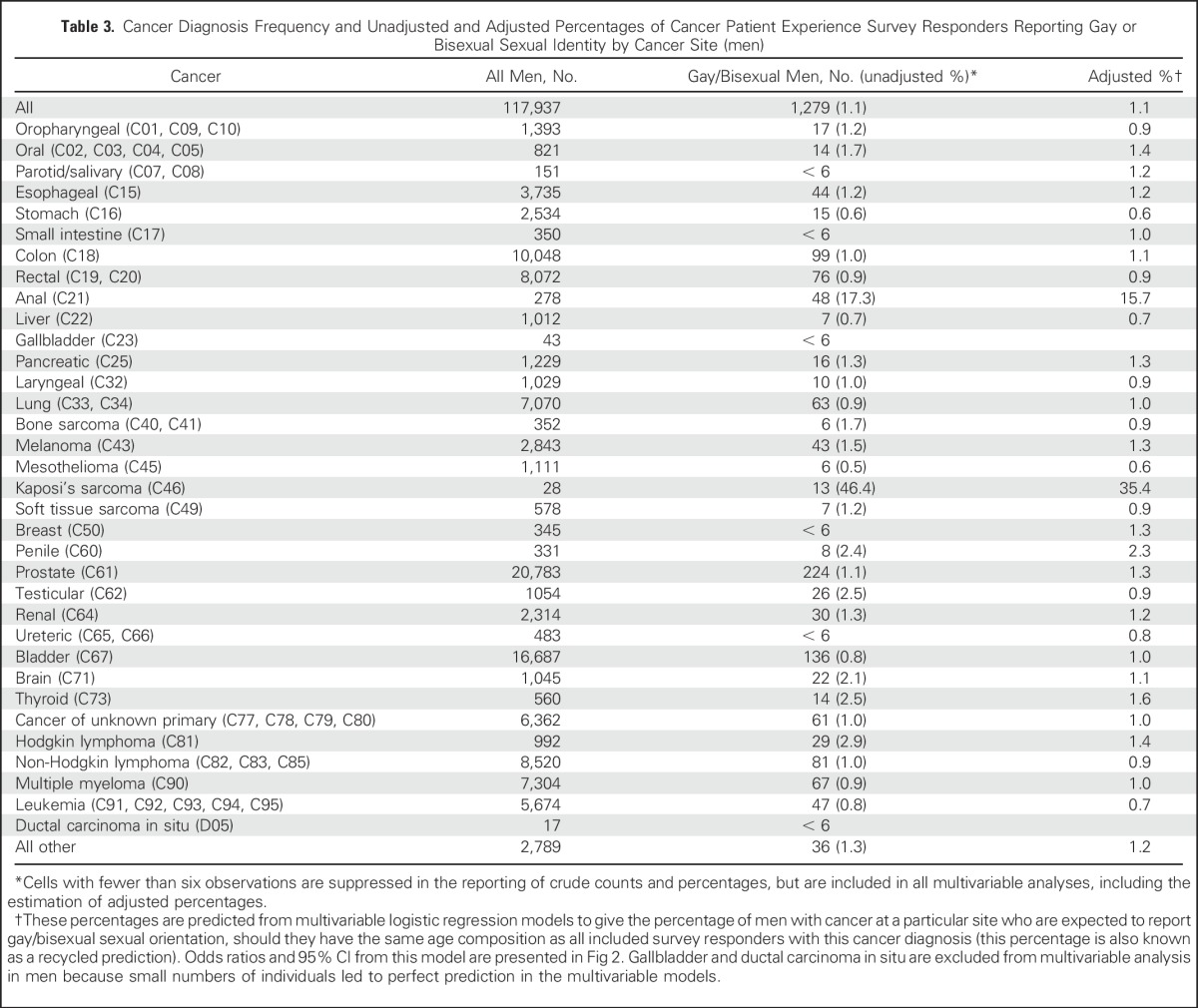
Cancer Diagnosis Frequency and Unadjusted and Adjusted Percentages of Cancer Patient Experience Survey Responders Reporting Gay or Bisexual Sexual Identity by Cancer Site (men)

**Fig 1.**
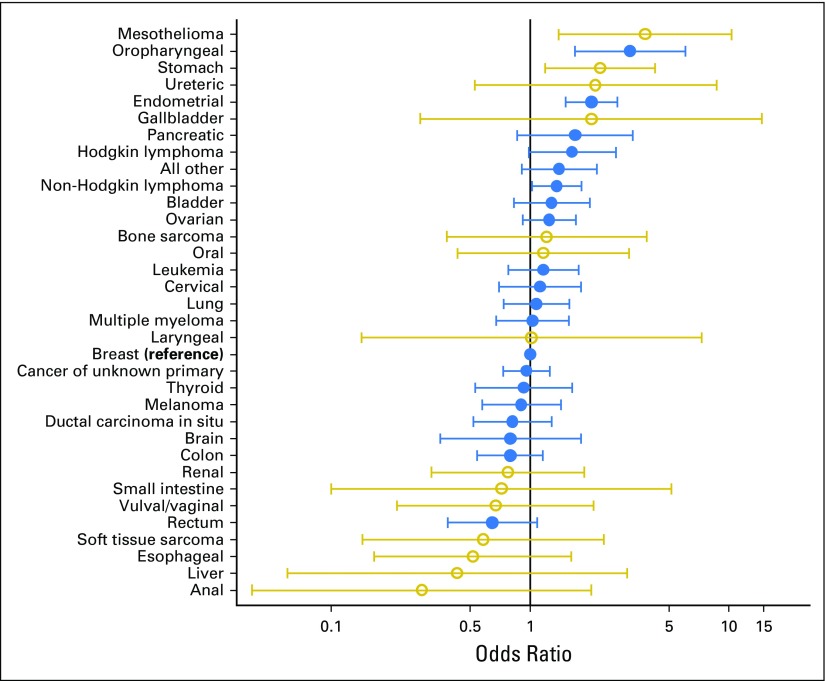
Odds ratios of specific cancer site diagnosis by lesbian or bisexual orientation among women with cancer, adjusted for age (Cancer Patient Experience Survey). Diagnoses represented with gold circles indicate fewer than six women with this diagnosis reporting lesbian or bisexual sexual identity (CPES); these diagnoses were included in the analysis model in the same way as other cancers, however the gold circles highlight that these results are based on relatively small numbers of cases.

**Fig 2.**
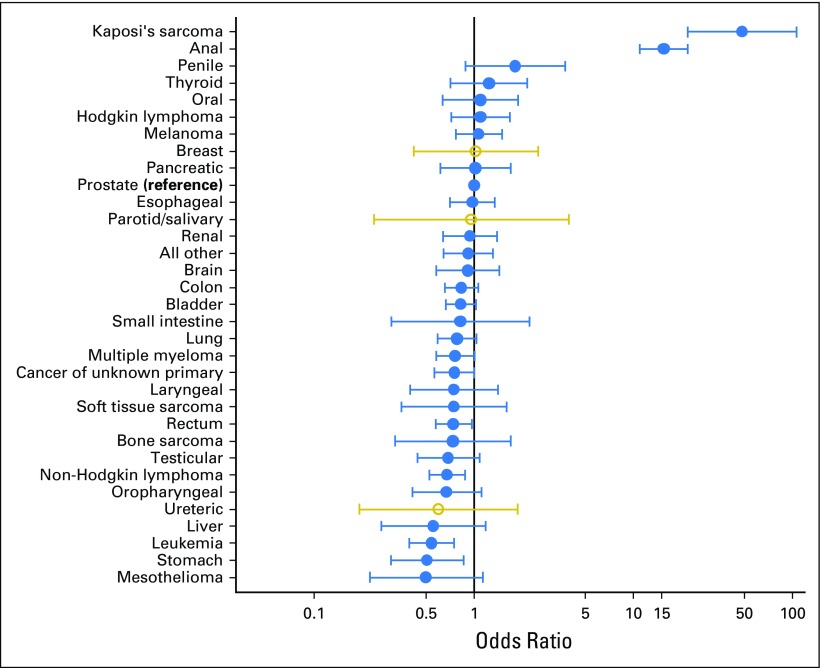
Odds ratios of specific cancer site diagnosis by gay or bisexual orientation among men with cancer, adjusted for age.

After adjusting for age, there continues to be statistical evidence that the proportion of people from sexual minorities varies between cancer sites (women, *P* = .0002; men, *P* < .0001). Whereas evidence for overall variation is statistically significant, the number of cases for many sites is small, which resulted in wide CIs (Figs 1 and 2). For most cancer sites that were examined in our adjusted analysis (30 of 33 in women, 28 of 32 in men), there was no evidence that sexual minorities were over- or under-represented compared with the most common cancers in each gender (female, breast; male, prostate); however, there were a few notable differences, including some infection-related (HIV or HPV) cancers. Lesbian/bisexual women are more frequently represented among women with oropharyngeal cancer (OR, 3.2; 95% CI, 1.7 to 6.0), and less frequently represented in anal (OR, 0.3; 95% CI, 0.0 to 2.0), and vulval/vaginal cancers (OR, 0.7; 95% CI, 0.2 to 2.1), although CIs are wide. Gay or bisexual men are relatively more frequently represented among men with Kaposi’s sarcoma (OR, 48.2; 95% CI, 22.0 to 105.6), anal (OR, 15.5; 95% CI, 11.0 to 21.9), and penile cancer (OR, 1.8; 9% CI, 0.9 to 3.7). In addition, lesbian or bisexual women are more frequently represented among women with mesothelioma, stomach, and endometrial cancers, but less among liver and esophageal cancers. Gay or bisexual men are more frequently represented among men with thyroid and oral cancers, melanoma, and Hodgkin lymphoma, and are relatively less frequently represented in liver and stomach cancers, leukemia, and mesothelioma.

In supplementary analyses, self-reported cancer prevalence among GPPS responders was lower among women with a missing response regarding their sexual orientation (*P* = .027) and men who stated, “Prefer not to say” (*P* < .001), with a small but significant (men, *P* = .0005; women, *P* = .001) variation in missing responses by cancer site, and “Prefer not to say” (men, *P* < .001). Considering respondents who had been diagnosed only in the past year, findings were consistent with the main analyses of CPES respondents. Variation in cancer diagnosis was also consistent when considering gay or lesbian and bisexual CPES responders separately (Appendix Tables A4 to A6, online only).

## DISCUSSION

We report large-scale evidence from two nationwide patient surveys in England, including information on self-reported sexual orientation, to explore whether women and men from sexual minorities report a cancer diagnosis in the previous 5 years more or less frequently than heterosexual women and men, and, among recently treated survivors of cancer, whether there is variation between cancer sites in the proportion of men and women who report gay, lesbian, or bisexual sexual orientation. We find that gay or bisexual men are more likely to have had cancer in the past 5 years than heterosexual men of the same age, although there was no evidence of a difference in cancer prevalence between heterosexual and lesbian or bisexual women. For both men and women, sexual orientation seems to be unrelated to the diagnosis of most cancer sites, particularly the more common cancers, but sexual minorities are over- or under-represented among patients of certain rarer sites. Cancer sites associated with HPV—and HIV—infection are those with the greatest degree of variation in the proportion of men and women from sexual minorities.

Regarding cancer prevalence, against limited overall evidence, our findings are consistent with a single other similar source, which also found similar cancer prevalence among heterosexual and sexual minority women, but increased odds among gay and bisexual men compared with heterosexual men.^35^ With regard to HPV-associated cancers, increased prevalence of anal cancer among gay men has been previously described^36^; however, to our knowledge, the relative over-representation of lesbian and bisexual women among women with oropharyngeal cancer has not previously been described and constitutes a novel finding. A history of performing oral sex is a known risk factor for oropharyngeal cancer.^37^ Previous evidence indicates that there is a higher probability of HPV transmission via vaginal oral sex compared with penile oral sex,^38,39^ which would result in a higher burden of oral HPV infection among lesbian and bisexual women.

Cervical cancer is associated with HPV infection, yet we do not find that lesbian or bisexual women are under-represented among women with cervical cancer. Our findings may be explained by a relatively low uptake of screening^40-42^ plus an earlier initiation of sexual intercourse.^43^ There have been inconsistent messages in the past about whether lesbian women need to attend cervical screening programs,^20,40^ but our data do not suggest any reduced need.

There are also some unexpected differences in cancer diagnosis by sexual orientation, in particular, the excess risk of mesothelioma and stomach cancer in lesbian women. Here, our findings may add novel insights, although with the caution that numbers are relatively low. Nonetheless, differences by sexual orientation among women with regard to occupational exposures, smoking (contributing to cancer risk across several sites), or dietary factors may be important, although this is speculative. The higher risk of endometrial cancer among lesbian or bisexual women is also surprising and is inconsistent with prior evidence.^44^

There are limitations that are inherent in all survey research, and, in this study, one such limitation is that the CPES analysis is based on treated patients, rather than population-based incident cases, and so rates cannot be estimated; however, our sensitivity analysis using only cases diagnosed in the last year—more similar to an incident population^34^—provided results that were consistent with the main analyses presented. Additional strengths of our study are its large analyses samples, the examination of both common and rare cancer sites, and the use of well-characterized national survey data.^25,45-48^

Although response rates for CPES are high (64% to 67%), response rates for GPPS were 37%; however, response rates alone are a poor indicator of bias.^49^ In addition, a randomized controlled trial demonstrated no variation in GPPS response rates when a question about the sexual orientation of the participant was included or excluded; this does not exclude, but greatly mitigates, the potential for survey nonparticipation bias by sexual orientation.^45^

We acknowledge that some people who identify as lesbian, gay, or bisexual may be unwilling to acknowledge their identity in a survey^50^; increased homelessness among young lesbian, gay, and bisexual people in the United Kingdom may also lead to additional under-representation among survey responders.^51^ In addition, item nonresponse is another concern, with people who do not respond to the sexual orientation question at all (in GPPS) possibly also being less likely to report any long-term conditions. People who report greater concerns about privacy are less likely to respond to sensitive demographic survey questions,^52^ and it is likely that the same mechanism may apply in our study context.

Sexual orientation and sexual behavior are different constructs; in this work, we consider cancer diagnosis associated with sexual orientation, although we acknowledge that HPV infection–associated cancer risk is primarily related to sexual behavior. The survey instruments encompassed heterosexual, gay, lesbian, and bisexual orientation, which is consistent with survey questions that were developed and validated by the UK Office of National Statistics^53^ that, however, do not encompass all sexual orientation and gender identity groups.

HPV vaccination presents an important opportunity for cancer prevention.^43^ In the United Kingdom, the current vaccination program covers girls age 12 to 13 years, and a recent pilot HPV vaccination schedule for men who have sex with men was rolled out in 2016.^54^ Modeling work is still in progress to decide whether all boys should receive the vaccination at age 12 to 13 years alongside girls.^55^ The research presented here provides additional epidemiologic evidence to inform decisions about the most equitable, effective, and cost-effective HPV vaccination schedules. Evidence from the United States suggests that HPV vaccination rates among lesbian women are low.^56^ Our research provides additional evidence that should particularly support efforts to encourage vaccination among lesbian and bisexual women.

This work presents population-based evidence about cancer prevalence among men and women from sexual minorities and about the relative frequencies of people from sexual minorities with common and rarer cancer diagnoses among recently treated survivors of cancer. Demographic data on cancer among people from sexual minorities are scarce^20^; these findings begin to address this evidential need. Finally, our research also highlights the importance of HPV vaccination among gay, lesbian, and bisexual women and men.

## Figures and Tables

**Table A1.**
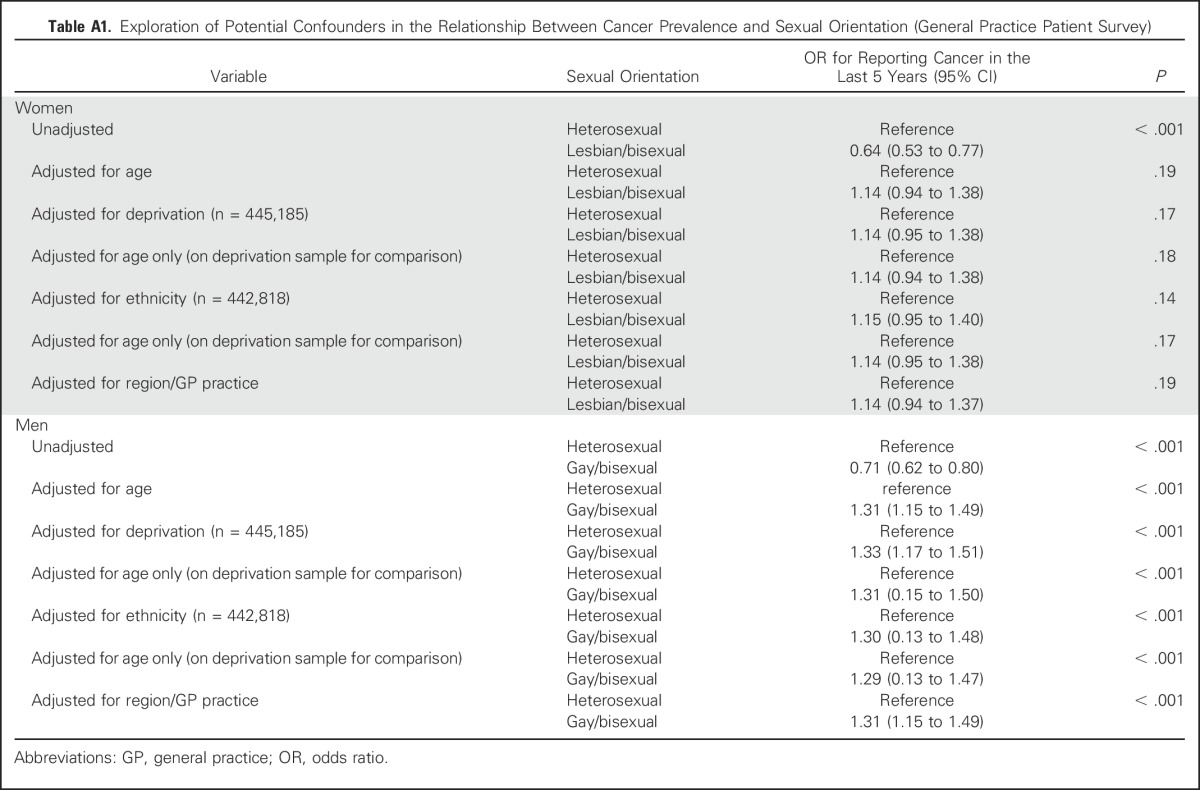
Exploration of Potential Confounders in the Relationship Between Cancer Prevalence and Sexual Orientation (General Practice Patient Survey)

**Table A2.**
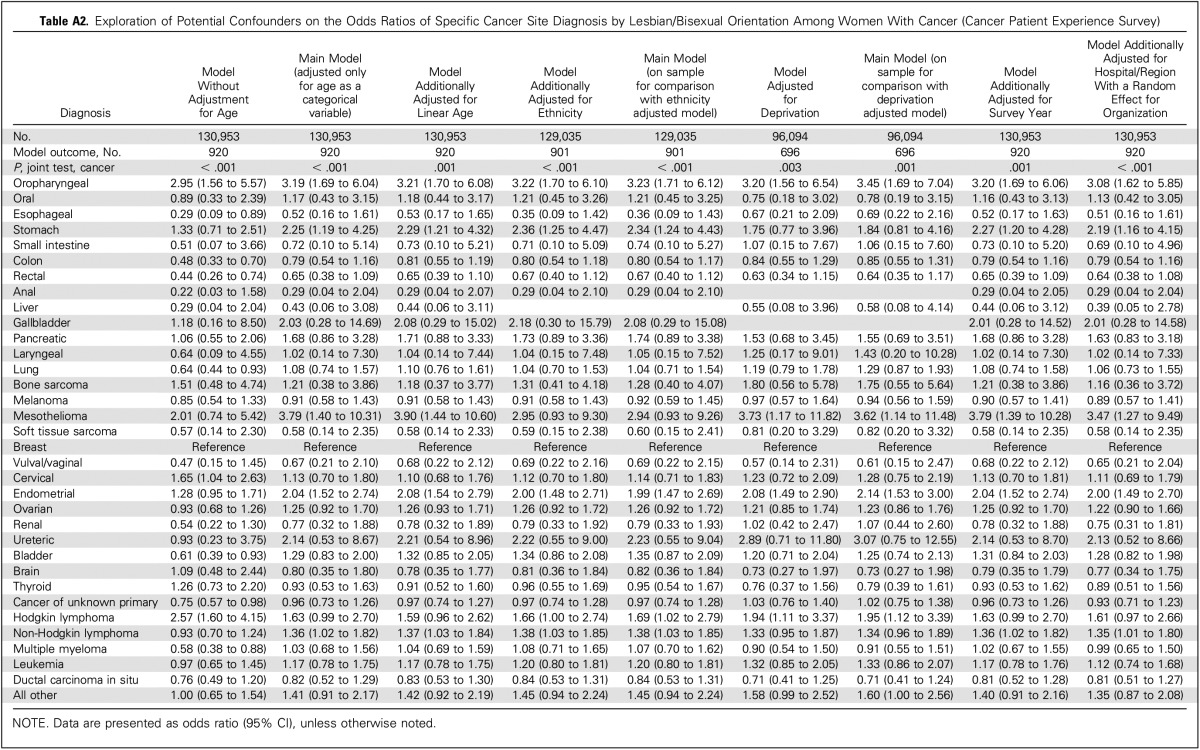
Exploration of Potential Confounders on the Odds Ratios of Specific Cancer Site Diagnosis by Lesbian/Bisexual Orientation Among Women With Cancer (Cancer Patient Experience Survey)

**Table A3.**
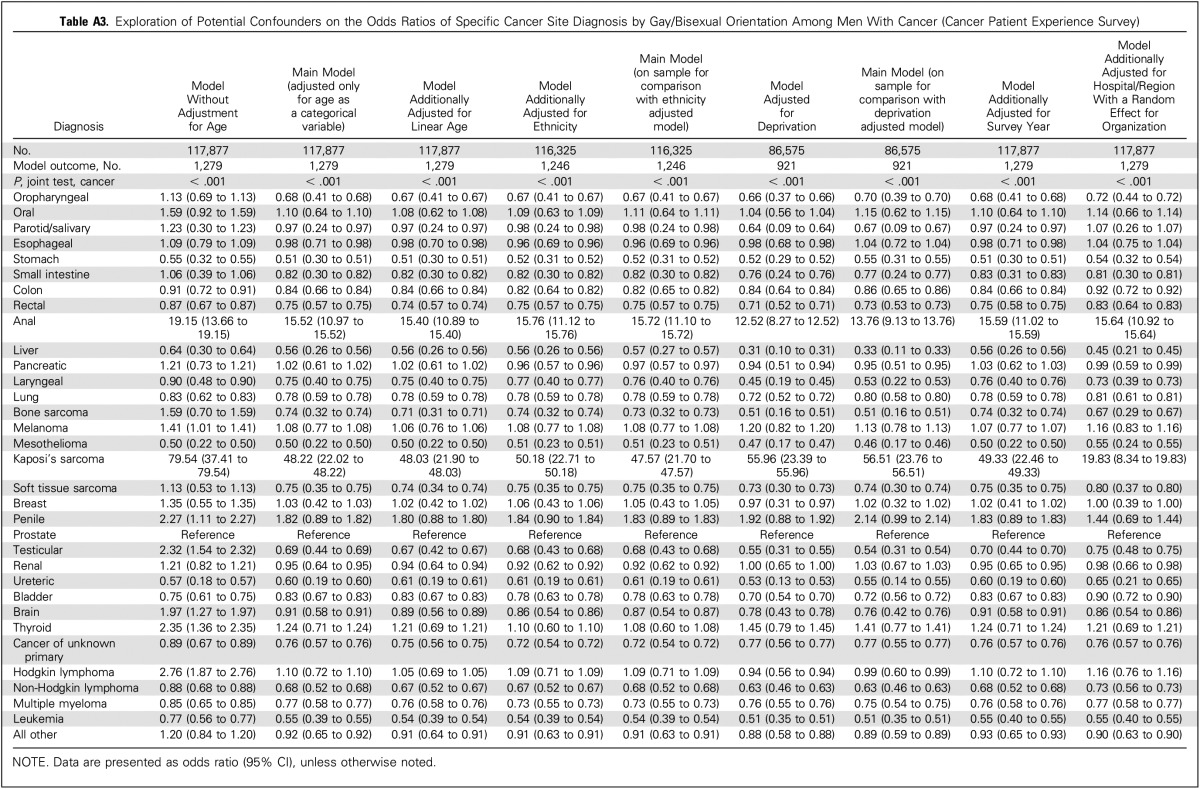
Exploration of Potential Confounders on the Odds Ratios of Specific Cancer Site Diagnosis by Gay/Bisexual Orientation Among Men With Cancer (Cancer Patient Experience Survey)

**Table A4.**
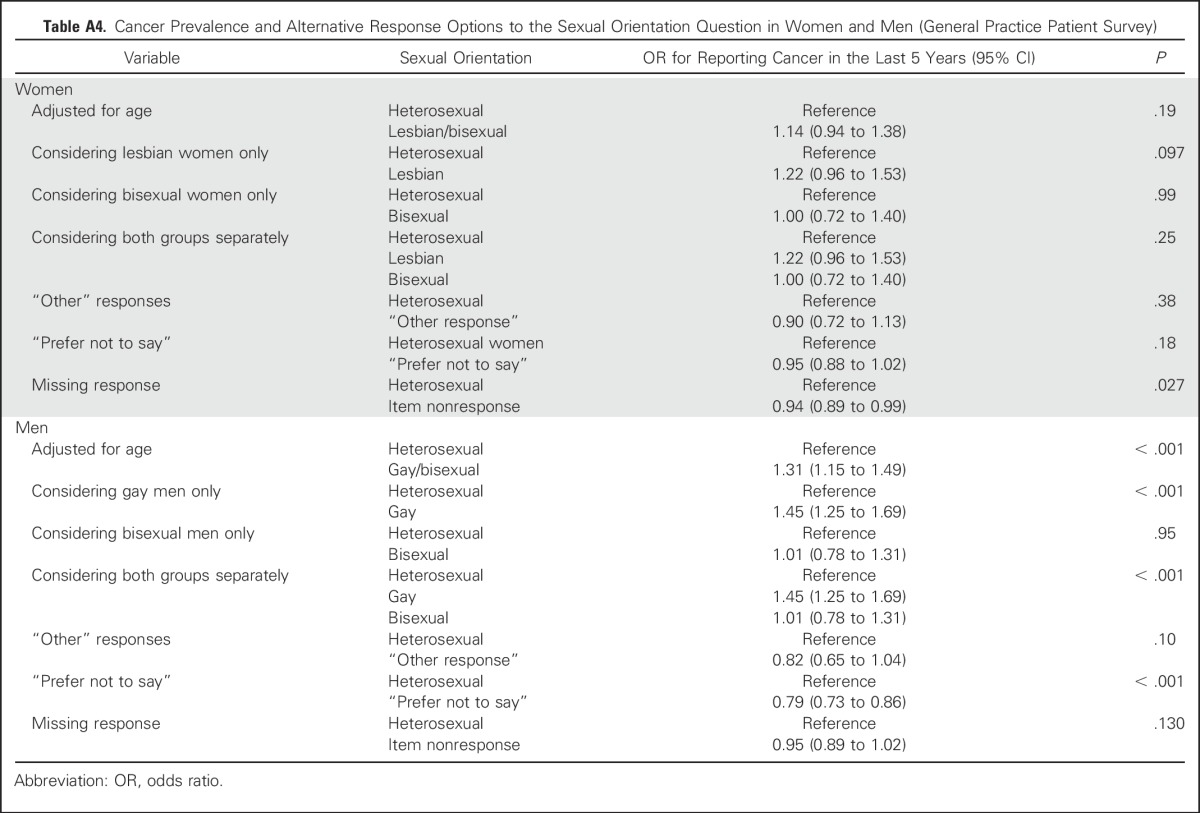
Cancer Prevalence and Alternative Response Options to the Sexual Orientation Question in Women and Men (General Practice Patient Survey)

**Table A5.**
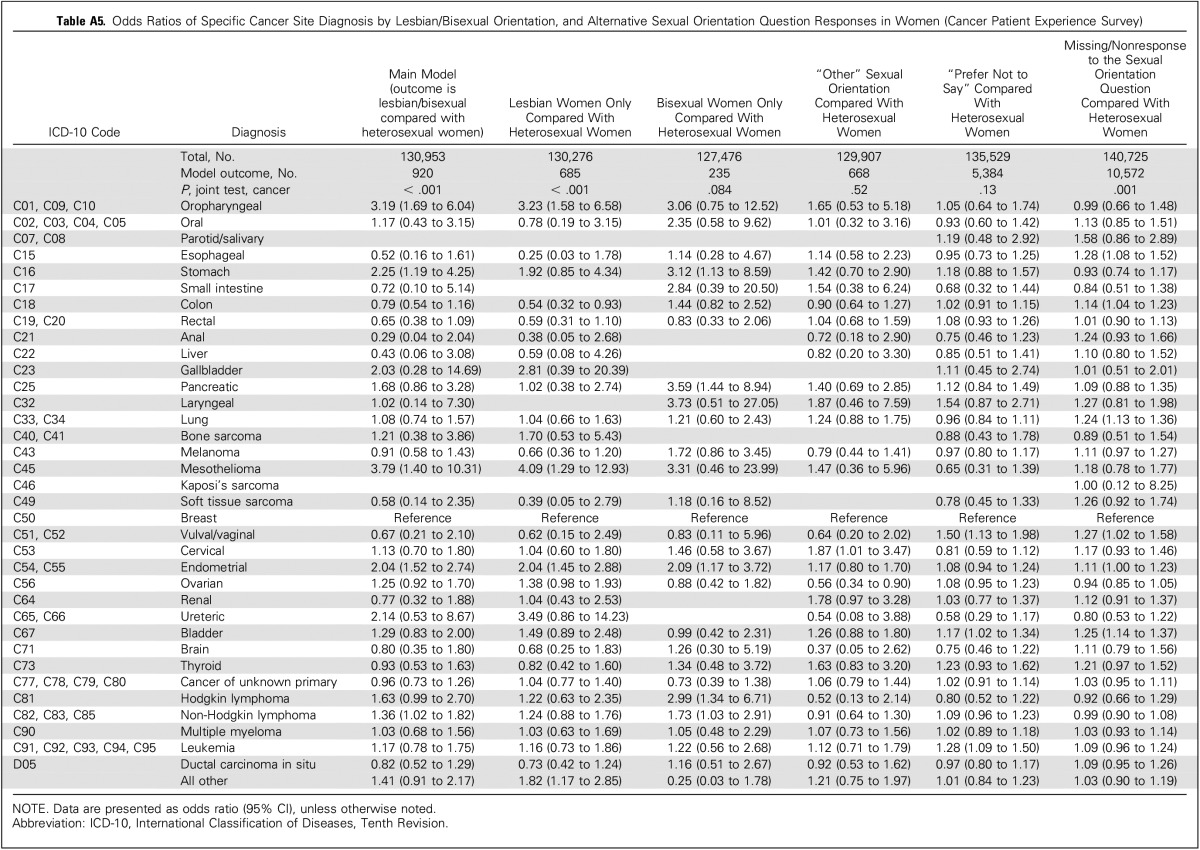
Odds Ratios of Specific Cancer Site Diagnosis by Lesbian/Bisexual Orientation, and Alternative Sexual Orientation Question Responses in Women (Cancer Patient Experience Survey)

**Table A6.**
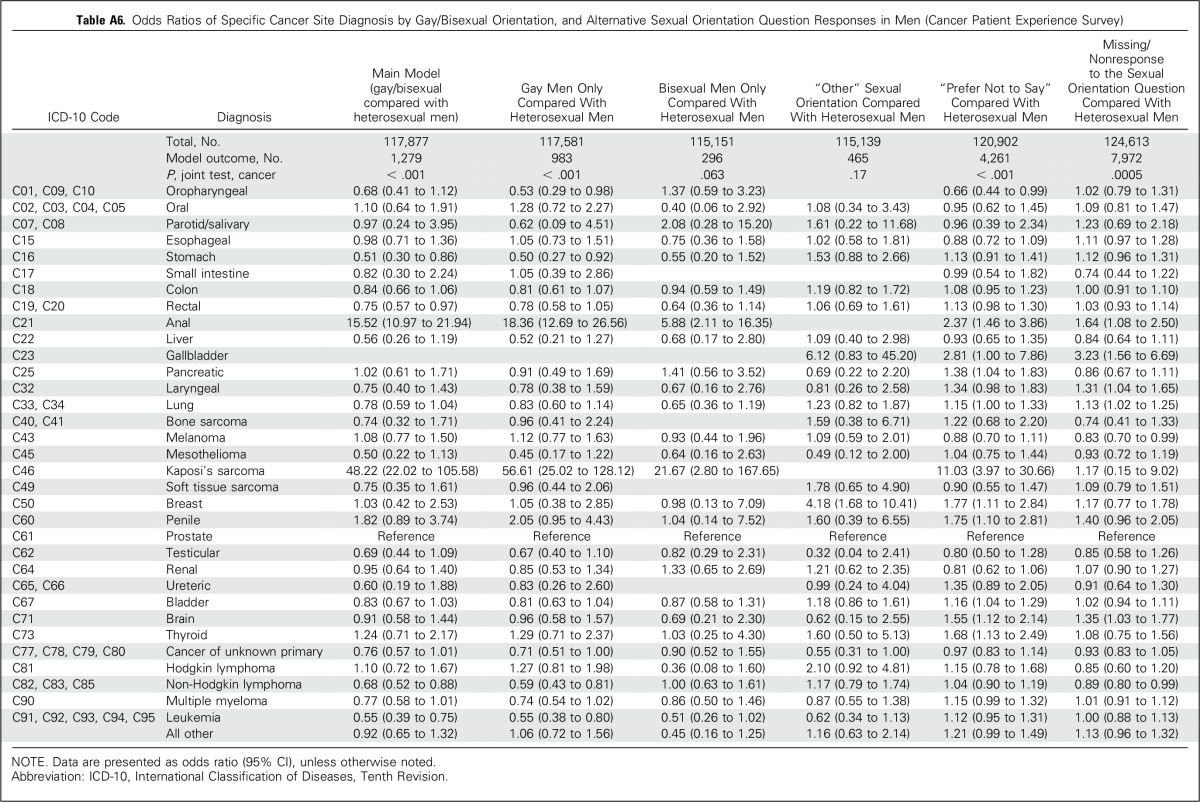
Odds Ratios of Specific Cancer Site Diagnosis by Gay/Bisexual Orientation, and Alternative Sexual Orientation Question Responses in Men (Cancer Patient Experience Survey)

**Table A7.**
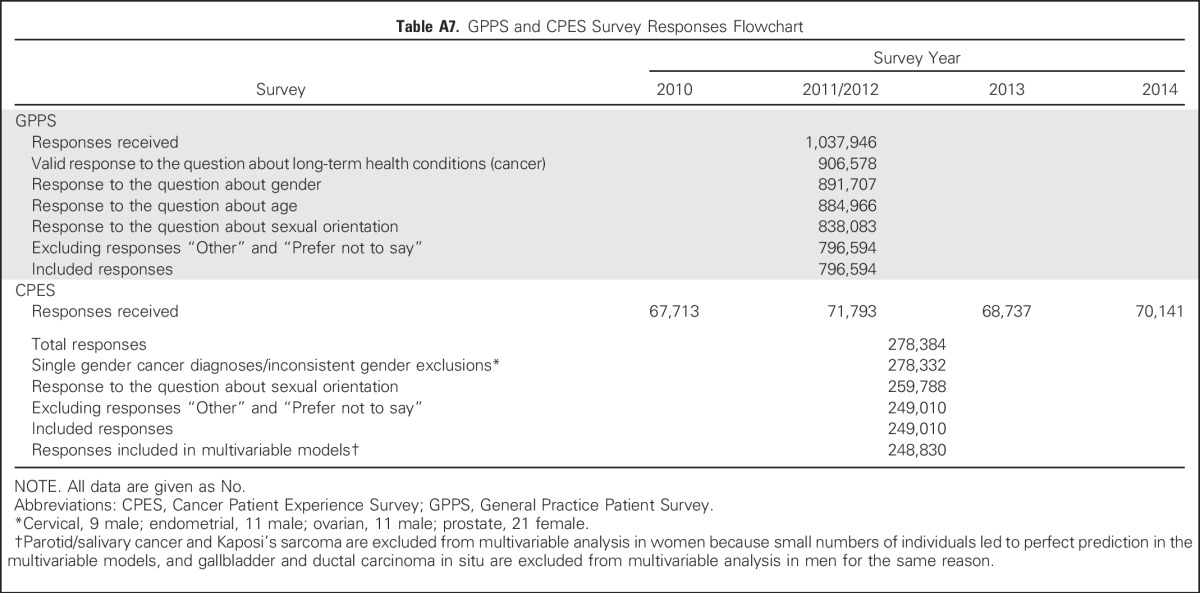
GPPS and CPES Survey Responses Flowchart

**Table A8.**
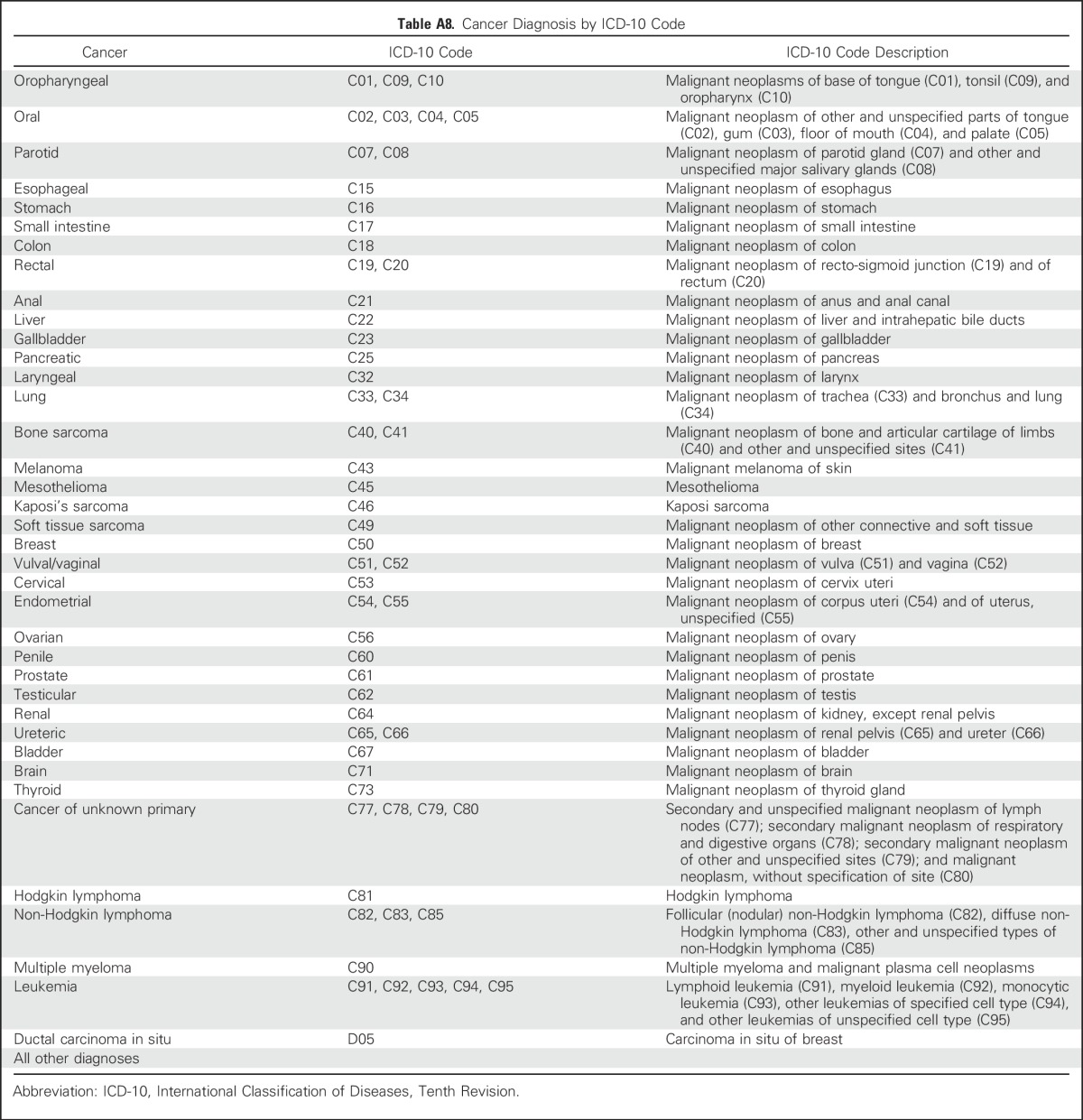
Cancer Diagnosis by ICD-10 Code

## Appendix

### Appendix Tables A1 to A3

In the main analyses presented in this article, we adjust only for age as a categorical variable. We also consider as possible confounders race and ethnicity, an additional age term, socioeconomic inequalities, survey wave (year), and hospital/general practice of treatment. As including these variables did not change the magnitude of the effect sizes of the association between sexual orientation and cancer diagnosis—results presented in Appendix Table A1 for General Practice Patient Survey (GPPS) and Appendix Tables A2 and A3 for Cancer Patient Experience Survey (CPES)—we did not include them in the final analysis models, which were presented only adjusted for age and stratified by sex. In addition, including additional covariates in our analysis models led to issues with smaller sample sizes—deprivation and ethnicity information is missing for some respondents—and issues of perfect prediction for some rarer cancer site diagnoses. Although the final analysis models are therefore parsimonious, unmeasured confounding is not expected to be a major concern.

The only coefficient that does change is that for Kaposi’s sarcoma in men after adjusting for hospital (reduction from 48.5 to 19.2; Appendix Table A3). Even after adjustment, this remains the cancer diagnosis with the strongest association with sexual orientation, and reflects the fact that patients with Kaposi’s sarcoma were only treated in a small number of hospitals overall, rather than more general confounding by region, and do not change the overall conclusions of this work. As we are interested in population-level estimates, rather than within-hospital estimates, in this analysis, we did not include this random effect in the final model.

### Appendix Tables A4 to A6

Survey questions were used to identify respondents’ sexual orientation in both surveys. In GPPS, “Which of the following best describes how you think of yourself?” had the following possible responses: “Heterosexual/straight,” “Gay/Lesbian,” “Bisexual,” “Other,” or “I would prefer not to say.” In CPES, “Which of the following best describes your sexual orientation?” had the following possible responses: “Heterosexual/straight (opposite sex),” “Bisexual (both sexes),” “Gay or Lesbian (same sex),” “Other,” or “Prefer not to answer.” To explore the sensitivity of our findings to different response options, we investigated the associations between each of the sexual orientation response options and the overall and site-specific diagnoses of cancer in a series of analyses. In particular, in the CPES analyses, the models for which the responses, “Bisexual” and “Gay” or “Lesbian,” are not grouped are consistent with the main analyses presented here, although CIs are wider, and variability in effect sizes are likely to reflect this imprecision.

### Appendix Table A5

Results from the main analysis model (presented in Fig 1) are presented in column 1 in this table. Additional analyses explore whether there is any variation between cancer sites in the proportion of women who report “Other” or “Prefer not to say” sexual orientation, or who do not respond to this question at all, and can be compared with the findings from the main model. Although there is evidence that the proportion of women who did not respond to the sexual orientation question varies between cancer sites, the magnitude of these differences are small (all odds ratios are close to 1), particularly compared with the variation in the main model

### Appendix Table A6

Although from these analyses there is evidence that the proportion of men who responded with the “Prefer not to say” option or who did not respond to the sexual orientation quesiont varies between cancer sites, the magnitude of these differences are small (all odds ratios are close to 1), particularly compared with the variation in the main model. Men with anal cancer have 2.4 times greater odds of giving a “Prefer not to say” response to the survey question about sexual identity than men with prostate cancer, and men with Kaposi’s sarcoma have 11.0 times greater odds of giving a “Prefer not to say” response. Clearly, there is some increased use of the “Prefer not to say” option among gay or bisexual men with these diagnoses, but it is not possible to determine the extent to which this option is used more frequently by all gay or bisexual men, or whether this is particularly related to these diagnoses that perhaps have greater stigma attached.

### Appendix Tables A7 and A8

These tables present details of the sample derivation and clinical coding of cancer sites used in the main analysis.

### Notes to Support the Comparison of Results From Tables 1 to 3

In Table 2, this analysis uses data from the GPPS to answer the research question, “Do women and men from sexual minorities report a cancer diagnosis in the previous 5 years more or less frequently than heterosexual women and men?”

In Tables 1 and 3, the CPES is used to explore the question, “Among recently treated survivors of cancer, is there variation between cancer sites in the proportion of men and women who report gay, lesbian, or bisexual sexual orientation?”

GPPS data are a population-based sample of the population of England, and we can therefore estimate cancer prevalence among men and women from sexual minorities—with and without cancer in the past 5 years—and compare this with people who report a heterosexual sexual orientation. This is a unique data source, as the large sample size—nearly 1 million people—and the availability of sampling and design weights allow us to estimate cancer prevalence in the last 5 years in England, stratified by sexual orientation, then adjusted for age. These results are presented in Table 2.

CPES data are also a unique survey resource of recently treated survivors of cancer, sent annually to all patients age ≥ 16 years. The strengths of this data resource are the large number of people with cancer—249,010 patients, including 920 lesbian or bisexual women and 1,279 gay or bisexual men (these numbers of people from sexual minorities with cancer are much higher compared with the population-based GPPS sample, which includes both people with and without cancer—and the availability of specific International Classification of Diseases, Tenth Revision, diagnosis codes, which are not available for the GPPS data; however, the two limitations in the CPES data are, first, the sample is hospital based, rather than population based (recently treated survivors of cancer), and, second, that we do not have a comparison population of people without cancer. This means that we cannot estimate cancer prevalence by using the CPES data. Instead, it is possible to estimate whether there is variation between cancer sites in the proportion of men and women who report gay, lesbian, or bisexual sexual orientation by using a case-only analysis.

These proportions allow us to explore whether people from sexual minorities are relatively over- or under-represented among people with a particular cancer diagnosis compared with baseline diagnosis—that is, are there more people from sexual minorities with a particular cancer site diagnosis than would be expected were there no variation between sites? This over- or under-representation will reflect higher or lower incidence, or higher or lower survival, among people from sexual minorities, but incidence and prevalence cannot be measured directly, and the comparison is therefore between cancer sites, rather than with people without cancer.

In practical terms, this means for the analysis that results in Tables 1 to 3 are complementary but not directly comparable, as the populations and the analyses are different. In Table 2, results tell us about cancer prevalence in the whole population of England (age ≥ 18 years)—that prevalence is higher among gay or bisexual men, but not among lesbian or bisexual women. In Tables 1 and 3, results tell us about variation between cancer sites, but not about comparisons with the whole population.
